# Enhancing post-thaw sperm quality in rams: quinic acid as a natural antioxidant

**DOI:** 10.3389/fvets.2025.1744161

**Published:** 2026-01-05

**Authors:** Barış Denk, Murat Kırıkkulak, Şükrü Güngör, Mehmet Fuat Gülhan, Muhammed Enes İnanç, Fatih Avdatek, Deniz Yeni, Umut Taşdemir

**Affiliations:** 1 Department of Biochemistry Afyonkarahisar, Faculty of Veterinary Medicine, Afyon Kocatepe University, Afyonkarahisar, Türkiye; 2 Department of Reproduction and Artificial Insemination, Faculty of Veterinary Medicine, Afyon Kocatepe University, Afyonkarahisar, Türkiye; 3 Department of Reproduction and Artificial Insemination, Faculty of Veterinary Medicine, Burdur Mehmet Akif Ersoy University, Burdur, Türkiye; 4 Department of Medicinal Aromatic Plants, Technical Sciences Vocational School, Aksaray University, Aksaray, Türkiye; 5 Department of Reproduction and Artificial Insemination, Faculty of Veterinary Medicine, Ankara University, Ankara, Türkiye

**Keywords:** antioxidant activity, DNA integrity, high mitochondrial membrane potential, oxidative stress, quinic acid, ram sperm cryopreservation

## Abstract

**Introduction:**

This study investigated the effects of quinic acid (QA) supplementation at different concentrations (Control, 50, 100, and 200 μg/mL) on the post-thaw quality of ram semen, with a focus on motility, DNA integrity, flow cytometric parameters, and oxidative status.

**Materials and methods:**

A total of 40 ejaculates collected from Ramlic rams were cryopreserved using Tris-based extenders containing QA. Post-thaw sperm quality was evaluated using Computer-Assisted Sperm Analysis (CASA), flow cytometry assays for viability, mitochondrial activity, and lipid peroxidation, and the single cell gel electrophoresis (COMET) analysis for DNA integrity. Oxidative status was assessed through measurements of TAS, TOS, MDA, and OSI.

**Results:**

QA supplementation at 100 μg/mL significantly improved total and progressive motility and enhanced key kinematic parameters compared with the control group (*p* < 0.05). Flow cytometry analyses showed that spermatozoa treated with 100 μg/mL QA exhibited higher viability (SYBR+; 81.54 ± 2.64%) and high mitochondrial membrane potential (HMMP; 26.98 ± 2.25%), along with reduced lipid peroxidation (BODIPY+; 35.72 ± 4.58%) relative to the control (*p* < 0.05). COMET assay results indicated that QA treatment, particularly at 100 μg/mL, decreased tail length and tail moment values, signifying reduced DNA fragmentation. Regarding redox balance, 100 μg/mL QA significantly enhanced total antioxidant status (TAS; 1.45 ± 0.01 μmol/L) and lowered oxidative stress index (OSI; 58.96 ± 2.44) compared to control (*p* < 0.001). However, the highest dose (200 μg/mL) increased malondialdehyde (MDA; 58.90 ± 0.17 nmol/mL) and total oxidant status (TOS; 11.20 ± 0.80 mmol/L), indicating a possible pro-oxidant effect at excessive concentrations.

**Conclusion:**

In conclusion, QA exerted dose-dependent protective effects on sperm motility, viability, HMMP, and DNA stability during cryopreservation. The optimal concentration (100 μg/mL) effectively mitigated oxidative stress and improved post-thaw semen quality, suggesting that QA could serve as a promising antioxidant and cryoprotective additive for enhancing the success of artificial insemination programs in rams.

## 1 Introduction

Artificial insemination (AI) is a fundamental biotechnological technique in animal husbandry, serving as a key tool for achieving genetic improvement, enhancing production efficiency, and optimizing the use of reproductive resources (1). In small ruminant breeding, the extensive applicability of AI is contingent upon the dependable preservation of semen via cryopreservation (2). However, during the freezing and thawing process, spermatozoa are exposed to a series of physicochemical stresses, including cold shock, ice crystal formation, and osmotic imbalance, along with an increase in reactive oxygen species (ROS) (3). These factors collectively induce significant structural and functional impairments in sperm cells, such as membrane lipid peroxidation, mitochondrial dysfunction, acrosomal damage, and DNA fragmentation (4). These mechanisms have been well documented in ram semen and are considered among the primary causes of cryopreservation failure (5).

The degree of cryosensitivity varies among species; however, the relatively low cholesterol-to-phospholipid ratio in the plasma membrane of ram spermatozoa makes these cells particularly susceptible to cold shock (6). This biochemical characteristic facilitates the disruption of membrane fluidity and integrity during the freeze–thaw cycle, consequently leading to a loss of motility and a reduction in fertilizing capability (7). The detrimental effects of increased ROS generation and the resulting oxidative stress during cryopreservation on sperm function have been reported (8). Lipid peroxidation disrupts membrane fluidity, while DNA damage compromises genetic integrity, thereby reducing fertilization success. In addition, a decrease in mitochondrial membrane potential impairs ATP production, negatively affecting motility. Therefore, antioxidant strategies have been proposed as a rational and effective approach to minimize cryo-induced damage in sperm cryopreservation protocols (9). In the literature, various enzymatic and non-enzymatic antioxidants (e.g., glutathione, vitamin E/C derivatives, and plant-derived phenolic compounds) added to semen extenders have been reported to mitigate motility loss, decrease DNA damage, and preserve acrosomal integrity and viability in frozen–thawed semen samples. Consequently, multiparametric methodologies, including Computer-Assisted Sperm Analysis (CASA)-based kinematic analysis, the Single cell gel electrophoresis assay (COMET) for DNA damage assessment, flow cytometric evaluation of functional sperm parameters, and biochemical redox markers, are essential for clarifying underlying mechanisms and formulating practical recommendations (10).

Quinic acid (QA) a cyclohexanecarboxylic acid derivative, is widely distributed in plants and food sources. Several experimental models have demonstrated its protective effects against oxidative stress-related conditions in recent years. Studies conducted on various animal models, including mice and rodents, as well as on the model organism *Caenorhabditis elegans* and cellular models of neurodegenerative diseases, have demonstrated that QA can reduce ROS levels, enhance cellular viability, and modulate stress response pathways. Such findings suggest that QA may influence intracellular stress regulatory mechanisms such as SKN-1/NRF2-like signaling pathways in addition to exerting direct antioxidant activity (11). Limited studies directly investigate the effects of QA on reproductive physiology, particularly in the context of semen cryopreservation. However, polyphenolic compounds structurally related to QA, such as chlorogenic acid, have been reported to exert cryoprotective and antioxidant effects on spermatozoa. Consequently, it is reasonable to suggest that QA may protect spermatozoa against oxidative damage through similar underlying mechanisms. In line with this, Noto et al. (12) demonstrated that chlorogenic acid reduced *in vitro* oxidative damage in human sperm and exhibited protective effects during cryopreservation. These findings suggest that QA may serve as a potential antioxidant or cytoprotective agent in animal and reproductive cells. However, it is well established that the effects of natural antioxidant compounds can be dose-dependent; while many exhibit antioxidant properties at low to moderate concentrations, they may shift toward pro-oxidant activity at higher doses, thereby disrupting redox homeostasis, which refers to the balanced regulation of cellular oxidants and antioxidants necessary for maintaining normal physiological functions. Therefore, it is crucial to evaluate QA across a range of concentrations using multiparametric approaches. Likewise, considering the specific sensitivities of ram semen and the distinct types of damage occurring during cryopreservation, a systematic assessment of QA in terms of motility/kinematic traits, DNA integrity, mitochondrial function, and redox markers is warranted (13).

This study aims to comprehensively evaluate the effects of different doses of QA on sperm motility and kinematic parameters, DNA damage, certain functional parameters (viability, lipid peroxidation, high mitochondrial membrane potential (HMMP), and redox balance Malondialdehyde (MDA), reduced glutathione (GSH), total antioxidant status (TAS), total oxidant status (TOS), and oxidative stress index (OSI)] in frozen–thawed ram semen. The ultimate goal of this research is to determine whether QA, at an appropriate concentration, can enhance post-thaw semen quality, and thereby demonstrating its potential as a novel cryoprotectant agent. To the best of our knowledge, this is the first study to investigate the effects of QA on the cryopreservation of ram spermatozoa.

## 2 Materials and methods

### 2.1 Semen collection and experimental design

Semen samples were collected from five Ramlic rams (a Daglic × Rambouillet crossbreed), aged between 2 and 3 years, using an artificial vagina at two-day intervals. The collection was conducted in Afyonkarahisar, Türkiye, during September–October, which corresponds to the region’s breeding season. In total, 40 ejaculates were obtained and assigned to experimental groups following a crossover design. Ethical approval for animal experimentation was granted by Afyon Kocatepe University (Ethics Committee Approval No: 49533702/169, March 13, 2024). Following collection, semen samples were immediately pooled to minimize the individual ram effect and then examined under a microscope. Ejaculates meeting the quality criteria of volume ≥0.6 mL, mass activity ≥+++3, motility ≥80%, and sperm concentration ≥2.0 × 10^9^/mL were used the study. A Tris-based extender was made with 3.63 g of Tris (T1503), 1.82 g of citric acid, and 0.5 g of fructose (F0127, C0759) per 100 mL of double-distilled water. It also had 15% egg yolk and 6% (v/v) glycerol. QA solutions were prepared fresh on the day of use. QA was first dissolved in 1 mL of the Tris-based extender to obtain a concentrated working stock at 200 μg/mL. This stock solution served as the source for all treatment groups and was not mixed directly with semen. Instead, defined volumes of the QA stock were added to the semen–extender mixture to achieve the final QA concentrations of 50, 100, and 200 μg/mL. All final concentrations were calculated based on the total post-dilution volume of semen and extender, ensuring the accurate attainment of the intended experimental doses.

The semen samples were separated into four equal parts and mixed with the Tris-based extender, which had control, 50 μg/mL, 100 μg/mL, and 200 μg/mL QA concentrations (C, QA50, QA100 and QA200 respectively). The diluted samples were equilibrated at +4 °C for 2 h, then loaded into 0.25 mL straws, frozen in liquid nitrogen vapor, and stored in liquid nitrogen at −196 °C. For subsequent analyses, frozen semen straws were thawed in a 37 °C water bath for 30 s.

### 2.2 Motility and kinetic characteristics

Sperm motility and kinematic parameters were analyzed using the CASA system (Sperm Class Analyzer, Microptic S.L., SCA® v.4.2, Spain) integrated with a phase-contrast microscope (Nikon Eclipse 50i, Japan). From each frozen–thawed semen straw, a 10 μL aliquot was placed on a microscope slide and covered with a coverslip. Evaluations were performed under a green-filtered, negative phase-contrast microscope at 100× magnification. Sperm cells were classified according to their curvilinear velocity as static (<10 μm/s), slow (10–45 μm/s), medium (45–75 μm/s), and rapid (>75 μm/s). A forward progressive movement rate of ≥75% was considered indicative of progressive motility. The assessed motility and kinematic parameters included total motility (%), progressive motility (Prog M, %), rapid (%), medium (%), slow (%), rapid progressive (R Prog, %), medium progressive (M Prog., %), non-progressive (N-Prog., %), curvilinear velocity (VCL, μm/s), straight-line velocity (VSL, μm/s), average path velocity (VAP, μm/s), amplitude of lateral head displacement (ALH, μm), beat-cross frequency (BCF, Hz), straightness (STR, %) [(VSL/VAP) × 100], linearity (LIN, %) [(VSL/VCL) × 100], and wobble (WOB, %) [(VAP/VCL) × 100]. For each sample, motility data were obtained by analyzing a total of 400 spermatozoa across five randomly selected microscopic fields.

### 2.3 Evaluations of flow cytometric analysis

**Figure 1:**
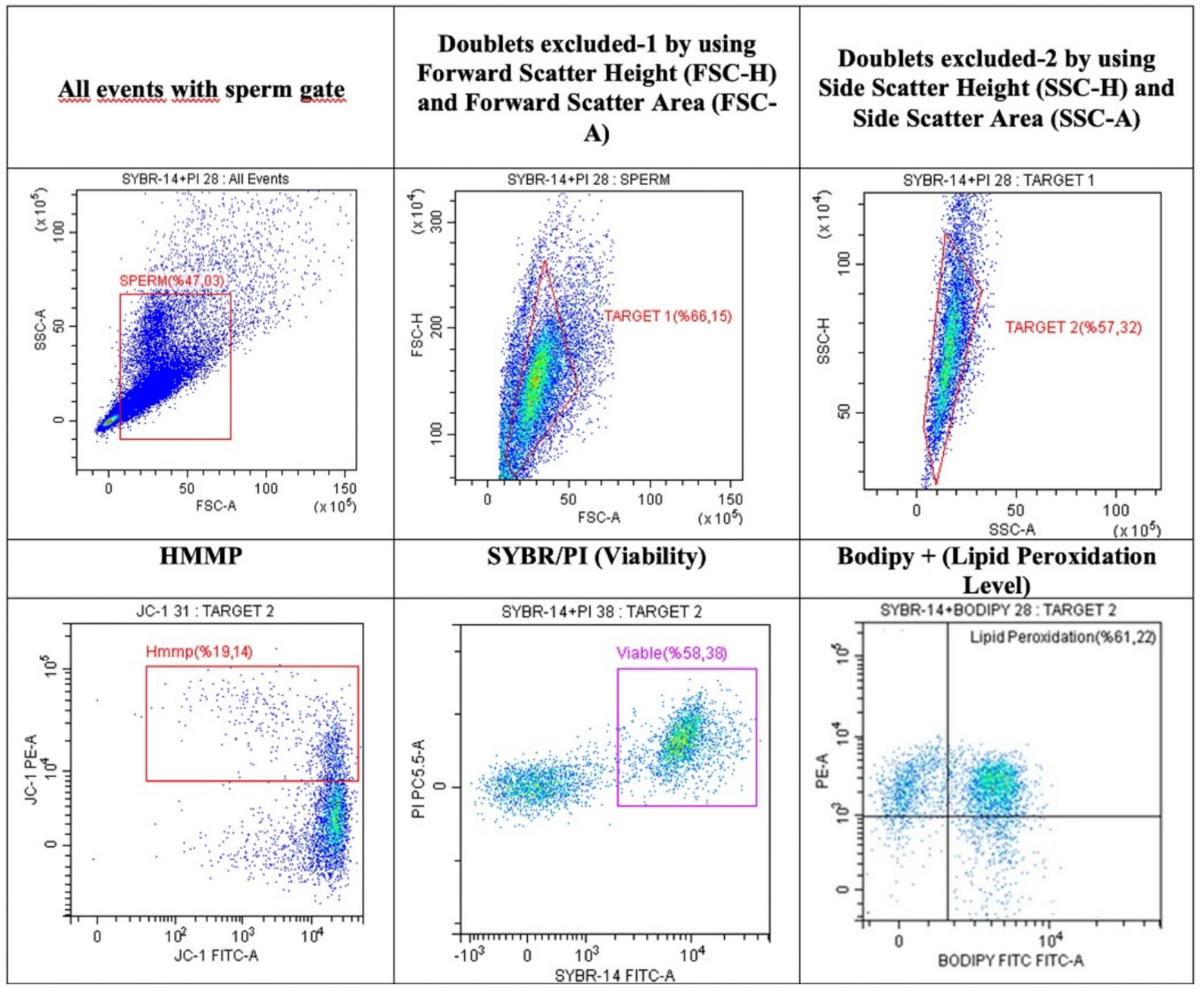
Flow cytometric assay dot plot images presented for the evaluation of sperm viability (SYBR+), lipid peroxidation (BODIPY+), and high mitochondrial membrane potential (HMMP) across experimental groups.

Flow cytometric analyses were conducted using a Beckman Coulter CytoFLEX flow cytometer (CA, USA), equipped with a 50 mW, 488 nm laser and emission filters of 525 ± 40 nm, 585 ± 42 nm, and 610 ± 20 nm. Approximately 10,000 spermatozoa were analyzed per sample. The forward scatter area (FSC-A) and side scatter area (SSC-A) signals were recorded to identify sperm populations, and pseudo-color plots were generated to compare FSC-A and FSC-H patterns. To exclude artifacts and debris, gating was applied based on the relationship between side scatter height (SSC-H) and SSC-A. For fluorescent staining, 50 μL aliquots were prepared using a dimethyl sulfoxide (DMSO) stock solution and stored at −20 °C until analysis. Flow cytometric analysis was evaluated using the CytExpert 2.3 software. High mitochondrial membrane potential (HMMP) was assessed using JC-1 dye (5,5′,6,6′-tetrachloro-1,1′,3,3′-tetraethylbenzimidazolylcarbocyanine). Sperm samples were diluted to 5 × 10^6^ spermatozoa/mL in PBS, after which 5 μL of JC-1 (emission of 629 nm, 0.153 mM, Molecular ProbesInvitrogen, T3168) was added to mixture and incubated for 15 min at 37 °C in the dark. HMMP values were then evaluated (14) Sperm viability was evaluated using SYBR-14 and PI double staining method. Sperm suspensions were adjusted to a final concentration of 5 × 10^6^ spermatozoa/mL in PBS, and then 5 μL SYBR-14 (1:10 diluation) and 3 μL PI (2,99 mM) were added final mixture. The mixture was incubated for 15 min at 37 °C in the dark. After incubation, viable (SYBR-14^+^) and dead (PI-) sperm populations were determined to viable (Figure 1). Spermatozoa lipid peroxidation levels were determined using BODIPY-SYBR staining according to the protocol described by Yeni et al. (15). The amount of spermatozoa was adjusted to a concentration of 5 × 10^6^ in 492 μL of PBS. Subsequently, 5 μL of BODIPY (5 μM, 519 nm, D38611, Molecular Probes, Invitrogen) and 3 μL of SYBR (1:10 dilution) were added, followed by incubation in a water bath at 37 °C for 15 min in a dark room. After the incubation period BODIPY+ and SYBR+ population were recorded as indicators of lipid peroxidation (Figure 1).

### 2.4 Evaluations of DNA fragmentations

Sperm DNA damage was evaluated using the the single cell gel electrophoresis (COMET) technique as outlined by Yeni et al. (15). The slides were analyzed using a fluorescent microscope (CX31, Olympus, Japan), and DNA fragmentation metrics, including tail length (TL, μm/s), tail moment (TM, μm/s), and tail DNA (%), were measured using COMET image analysis software (TriTek, version 1.5). A total of 200 spermatozoa were assessed for each sample over five randomly chosen microscopic areas.

### 2.5 Redox markers

A commercial colorimetric assay kit (Rel-Assay Diagnostics, Gaziantep, Türkiye) was employed to determine TOS and total antioxidant status TAS. In the TAS assay, antioxidants present in the samples reduce the dark blue-green colored 2,2′-azino-bis(3-ethylbenzothiazoline-6-sulfonic acid) radical cation (ABTS•^+^) to its colorless form. The decrease in absorbance measured at 660 nm correlates with the total antioxidant capacity of the sample, and the results are expressed as μmol/L. Trolox was used as the standard antioxidant for TAS calibration. In the TOS procedure, the color intensity generated by oxidizing agents in the samples was used to assess total oxidant status. The oxidation of ferrous ions (Fe^2+^) to ferric ions (Fe^3+^) by oxidants in the test system was quantified spectrophotometrically at 660 nm, with results also expressed as μmol/L. Hydrogen peroxide (H₂O₂) was used as the standard oxidant for TOS. The OSI was calculated using the formula OSI = [TOS / (TAS × 100)], following the method described by Esen et al. (16). MDA and lipid peroxidation levels were determined according to the procedure of Draper and Hadley (17). In this assay, MDA was quantified as nmol/mL, and lipid peroxidation was expressed as mmol/L, based on the formation of an MDA–thiobarbituric acid complex and its absorbance at 532 nm. A 1,1,3,3-tetramethoxypropane (TMP) standard was used for MDA quantification. GSH content was measured spectrophotometrically at 412 nm according to the method of Hilf and Hissin (18) and expressed as mg/dL. Reduced glutathione was used as the standard compound for calibration in the GSH assay. All reagents were obtained from Sigma-Aldrich (St. Louis, MO, USA), and double-distilled water was used for the preparation of all solutions.

### 2.6 Statistical analyses

Statistical analyses were performed using IBM SPSS Statistics software (v13.0, IBM Corp., Armonk, NY, USA). The Shapiro–Wilk test was used to look at the data’s distribution characteristics. For variables showing a normal distribution, one-way analysis of variance (ANOVA) was applied, and post-hoc tests were conducted to determine the differences between groups. Homogeneity of variances was assessed by Levene’s test; when homogeneity was not satisfied, Tamhane’s T2 test was used, whereas Duncan’s test was preferred when homogeneity was met. In all statistical evaluations, a significance level of *p* < 0.05 was considered.

## 3 Results

### 3.1 Sperm motility and kinematic parameters

QA supplementation markedly influenced several sperm motility and kinematic parameters in frozen–thawed ram semen (Table 1). Prog M significantly increased in QA100 (20.85 ± 2.46%) and QA200 (21.94 ± 3.25%) compared with the control [12.35 ± 2.09%; (*p* < 0.05)]. Similarly, the percentage of rapid motility was higher in the QA100 (8.09 ± 1.61%) and QA200 (9.65 ± 1.60%) groups than in the control (*p* < 0.01). R Prog also increased significantly in these groups (*p* < 0.01). Among kinematic parameters, VCL, VAP, and VSL values showed a dose-dependent increase, with the highest values recorded in the QA200 group (*p* < 0.05). Conversely, total motility, medium and slow motility, as well as LIN, STR, WOB, ALH, and BCF, did not differ significantly among groups (*p* > 0.05).

**Table 1:** CASA-based evaluation of sperm motility and kinematic parameters in frozen–thawed ram semen treated with different concentrations of QA.

Parameters	Control	QA50	QA100	QA200	*p*
PROG M (%)	12.35 ± 2.09^c^	14.08 ± 1.95^bc^	20.85 ± 2.46^ab^	21.94 ± 3.25^a^	**0.029**
MOTIL (%)	45.67 ± 6.12	46.14 ± 6.28	51.45 ± 3.98	48.22 ± 6.42	0.891
RAPID (%)	2.77 ± 0.61^b^	4.66 ± 0.94^b^	8.09 ± 1.61^a^	9.65 ± 1.60^a^	**0.004**
MEDIUM (%)	13.37 ± 2.01	14.44 ± 1.90	17.32 ± 1.49	16.63 ± 2.64	0.496
SLOW (%)	29.57 ± 4.02	27.04 ± 4.03	26.03 ± 3.27	21.93 ± 3.40	0.543
R PROG (%)	1.80 ± 0.42^b^	2.24 ± 0.49^b^	5.49 ± 1.25^ab^	6.61 ± 1.43^a^	**0.006**
M PROG (%)	10.55 ± 1.70	11.83 ± 1.50	15.36 ± 1.49	15.32 ± 2.05	0.137
N PROG (%)	33.31 ± 4.75	32.06 ± 5.08	30.60 ± 3.55	26.28 ± 4.91	0.727
VCL (μm/s)	49.97 ± 1.47^c^	53.87 ± 1.58^bc^	58.41 ± 2.14^ab^	62.02 ± 1.61^a^	**<0.001**
VAP (μm/s)	32.81 ± 1.85^c^	36.16 ± 2.14^bc^	39.97 ± 2.35^ab^	44.04 ± 1.96^a^	**0.007**
VSL (μm/s)	26.85 ± 1.93^b^	29.29 ± 2.41^ab^	33.04 ± 2.36^ab^	36.43 ± 2.54^a^	**0.042**
STR (%)	76.14 ± 1.46	76.23 ± 2.54	76.32 ± 1.67	75.07 ± 2.15	0.967
LIN (%)	52.31 ± 2.35	53.87 ± 3.21	54.19 ± 2.09	55.18 ± 2.78	0.894
WOB (%)	65.42 ± 1.92	67.42 ± 2.18	67.50 ± 1.54	69.24 ± 1.95	0.584
ALH (μm)	2.07 ± 0.09	2.06 ± 0.05	2.13 ± 0.06	2.18 ± 0.11	0.723
BCF (Hz)	10.38 ± 0.30	10.29 ± 0.48	10.72 ± 0.53	10.14 ± 0.47	0.838

Comparison of sperm motility and kinematic parameters (mean ± SEM) among the control and QA-treated groups (50, 100, and 200 μg/mL). Prog. M., parameters include progressive motility, Motil, motility, R Prog, rapid progressive, M Prog, medium progressive, N-Prog, Non-Progressive, VCL, curvilinear velocity, VAP, average path velocity, STR, straightness, LIN, linearity, WOB, wobble, ALH, amplitude of lateral head displacement, and BCF, beat frequency.

a, b: Different superscripts within the same row indicate significant differences (*p < 0.05). *p*-values represent the overall treatment effect (one-way ANOVA), and superscript letters denote significant *post-hoc* pairwise differences.

### 3.2 DNA damage parameters

The COMET assay revealed significant differences in sperm DNA integrity among groups treated with varying concentrations of QA (Table 2). Tail Length significantly decreased in the QA50 and QA100 compared to the control (*p* < 0.001), suggesting reduced DNA fragmentation. Similarly, tail DNA% was markedly lower in the QA200 than in the control. Tail moment values, another indicator of DNA strand breaks, were significantly reduced in the QA50 and QA100 (*p* < 0.001). These results demonstrate that QA, particularly at moderate doses (50–100 μg), effectively protects spermatozoa DNA from oxidative damage during cryopreservation.

**Table 2:** COMET assay results showing DNA damage parameters in frozen–thawed ram semen treated with different concentrations of QA.

Parameters	Control	QA50	QA100	QA200	*p*
Tail length (μm)	22.84 ± 0.95^a^	19.03 ± 0.62^b^	18.73 ± 0.69^b^	24.69 ± 1.02^a^	**<0.001**
Tail DNA (%)	38.27 ± 1.23^a^	38.12 ± 1.04^a^	36.35 ± 1.21^ab^	34.49 ± 1.22^b^	**0.035**
Tail moment	17.88 ± 0.53^a^	15.08 ± 0.58^b^	13.44 ± 0.59^b^	18.45 ± 0.93^a^	**<0.001**

Tail length, tail DNA percentage, and tail moment values (mean ± SEM) measured in control and QA-treated groups using the comet assay.

a, b: Different superscripts within the same row indicate significant differences (*p < 0.05). *p*-values reflect the overall group effect (one-way ANOVA), and superscript letters denote significant pairwise differences identified by post-hoc testing.

### 3.3 Redox parameters

QA treatments caused notable alterations in oxidative stress and antioxidant balance (Table 3). Treatment with QA100 resulted in increased TAS without a significant change in OSI, whereas the highest dose (QA200) led to elevated MDA and TOS levels, accompanied by a marked increase in OSI (*p* < 0.001). The QA200 enhanced lipid peroxidation in sperm, indicating insufficient antioxidant defense, whereas low and moderate doses (Qa50 and QA100) did not elevate MDA levels, suggesting preserved oxidative balance at these concentrations (*p* < 0.001). No significant differences were observed in GSH levels among groups (*p* > 0.05). TAS reflects the cumulative activity of all plasma antioxidants. QA at low and moderate doses enhanced antioxidant capacity, while TAS at QA200 returned to the control level rather than decreasing below it, indicating that excessive QA did not further enhance antioxidant defenses (*p* < 0.001). TOS, representing the total oxidant load, increased markedly at high QA doses, indicating pro-oxidant effects, whereas limited changes in the QA50 and QA100 suggest maintained redox balance.

**Table 3:** Effects of QA treatments on oxidative stress and antioxidant parameters (MDA, GSH, TAS, TOS, and OSI) in frozen–thawed ram semen.

Parameters	Control	QA50	QA100	QA200	*p*
MDA (nmol/mL)	54.48 ± 0.78^b^	55.46 ± 0.75^b^	55.81 ± 0.80^b^	58.90 ± 0.17^a^	**<0.001**
GSH (mg/dL)	45.75 ± 4.13	44.14 ± 3.87	42.47 ± 4.80	44.83 ± 3.86	0.816
TAS (μmol/L)	0.90 ± 0.01^b^	1.41 ± 0.01^a^	1.45 ± 0.01^a^	0.90 ± 0.02^b^	**<0.001**
TOS (mmol/L)	5.51 ± 0.88^c^	7.07 ± 0.31^bc^	8.60 ± 0.36^b^	11.20 ± 0.80^a^	**<0.001**
OSI (TOS/TAS × 100)	60.59 ± 9.71^b^	49.92 ± 2.06^b^	58.96 ± 2.44^b^	124.41 ± 9.50^a^	**<0.001**

TOS, total oxidant status; TAS, total antioxidant status; OSI, oxidative stress index; MDA, malondialdehyde; GSH, reduced glutathione values (mean ± SEM) for each group.

a, b: Different superscripts within the same row indicate significant differences (*p < 0.05). *p*-values represent the overall group effect (one-way ANOVA), and superscript letters denote significant pairwise differences identified through *post-hoc* testing.

### 3.4 Flow cytometric parameters

Flow cytometry revealed that QA supplementation influenced several functional sperm characteristics (Table 3). The proportion of viable sperm (SYBR+) significantly increased in QA100 (81.54 ± 2.64%) relative to the control group [70.95 ± 3.69%; (*p* < 0.05)]. Lipid peroxidation levels (BODIPY+) decreased markedly in QA100 (35.72 ± 4.58%) compared to the control (58.24 ± 3.21%; *p* = 0.022), indicating an antioxidant protective effect. However, the proportion of sperm with HMMP significantly increased in QA100 (26.98 ± 2.25%; *p* < 0.001), suggesting improved mitochondrial function and energy production. Collectively, these data suggest that QA at 100 μg optimally supports sperm viability and mitochondrial activity while minimizing lipid peroxidation level (see Table 4).

**Table 4:** Functional sperm parameters evaluated by flow cytometry in control and QA–treated groups.

Parameters	Control	QA50	QA100	QA200	*p*
SYBR + (%)	70.95 ± 3.69^b^	77.70 ± 3.28^ab^	81.54 ± 2.64^a^	73.96 ± 2.24^ab^	**0.028**
BODIPY + (%)	58.24 ± 3.21^a^	48.87 ± 5.97^ab^	35.72 ± 4.58^b^	50.88 ± 4.57^a^	**0.022**
HMMP (%)	19.46 ± 1.75^b^	21.44 ± 2.66^ab^	26.98 ± 2.25^a^	19.01 ± 1.58^b^	**<0.001**

Flow cytometric assessment of sperm lipid peroxidation (BODIPY+), viability (SYBR+), and high mitochondrial membrane potential (HMMP) in all groups (mean ± SEM).

a, b, c: Different superscripts within the same row indicate significant differences (*p < 0.05). *p*-values represent the overall treatment effect (one-way ANOVA), and superscript letters denote significant pairwise differences identified by *post-hoc* analysis.

## 4 Discussion

Motility and kinematic are regarded as an indispensable tool in reproductive biotechnology and semen quality research. These parameters are considered fundamental indicators of sperm fertilizing potential. They provide a more in-depth look at sperm functional competence after cryopreservation or other treatments than traditional microscopic assessments (19). The motility conducted in this study demonstrated that the addition of QA, particularly at 100 and 200 μg, significantly enhanced sperm motility and kinematic parameters, including Prog M, VCL, VAP, and VSL compared to the control. Overall, these findings indicate that QA enhances sperm forward motility and velocity in a concentration-dependent manner, particularly at 100–200 μg. This effect is likely attributed to the antioxidant properties of QA, which preserve membrane integrity and support flagellar movement. The dose-dependent rise in VCL signifies that spermatozoa demonstrate more robust and extensive flagellar oscillations, implying that QA may preserve mitochondrial function and consequently facilitate energy production. The gradual increase in VAP observed up to the QA200 group implies that QA contributes to a more linear sperm trajectory, potentially through the maintenance of membrane fluidity. Additionally, the significant enhancement in VSL in the QA100 and QA200 groups indicates improved linear progression, a critical biophysical parameter directly related to fertilizing potential.

These findings are consistent with previous studies reporting that antioxidant supplementation mitigates motility loss and structural damage during cryopreservation. Zhu et al. (20) reported that resveratrol supplementation improved motility, mitochondrial function, and kinematic parameters in frozen–thawed ram spermatozoa. Additionally, quercetin and other phenolic antioxidants have demonstrated positive effects on post-thaw sperm quality in bucks (21) and bulls (22). In harmony with our work, chlorogenic acid, a polyphenolic compound structurally related to QA, has been demonstrated to improve sperm motility and membrane stability at optimal dosages both ram (23) and human (12). These findings support the hypothesis that QA may exert cryoprotective effects through similar antioxidant mechanisms. This fact is consistent with the observed increase in oxidative stress markers in the QA200 group of the present study. Overall, the motility findings of this study align with previous research on phenolic antioxidants, suggesting that moderate QA supplementation, particularly at 100 μg, may serve as an effective cryoprotective agent by preserving sperm motility and functional integrity post-cryopreservation. Nonetheless, these effects require further validation at the molecular level and under *in vivo* fertility conditions.

In the present study, supplementation with QA100 significantly reduced tail length and tail DNA in spermatozoa compared to the control group (*p* < 0.001). These findings suggest that QA mitigates oxidative DNA damage incurred during the freeze–thaw process, thereby preserving sperm DNA integrity. Tail lenght, a well-established indicator of DNA strand breaks, was notably decreased by QA at low (50 μg/mL) and moderate (100 μg/mL) doses. However, according to tail lenght, and tail moment results, high-dose supplementation (200 μg/mL) appeared to increase DNA damage, likely due to disruption of the antioxidant–pro-oxidant balance. This observation indicates a dose-dependent biphasic effect of QA, providing protective benefits at low and moderate concentrations while potentially inducing oxidative stress at higher levels. The tail moment value is a critical indicator reflecting both the extent and amount of DNA damage. The observed reduction in tail moment in the QA50 and QA100 groups supports the protective effect of QA on sperm DNA integrity. Conversely, the increase in the QA200 group suggests potential adverse effects associated with higher doses. Although some parameters showed a dose-related tendency, no statistical trend analysis was performed; therefore, these patterns should be interpreted cautiously rather than as a confirmed linear trend. Based on these findings, the optimal effective dose range appears to be 50–100 μg. Structurally related polyphenols, such as chlorogenic acid, have also been reported to preserve DNA integrity after cryopreservation. Noto et al. (12) demonstrated that chlorogenic acid significantly improved tail DNA and tail moment in human sperm, an effect attributed to reduced ROS levels. Similarly, Zhu et al. (20) reported that resveratrol supplementation-maintained DNA integrity in ram sperm, while Avdatek et al. (24) observed that quercetin reduced DNA fragmentation in post-thaw bull sperm. These studies collectively support the notion that the DNA-protective effect of QA is mediated through antioxidant mechanisms. QA’s protective action is likely associated with the prevention of ROS-induced base modifications and double-strand breaks, thereby preserving DNA integrity. Moreover, QA may enhance endogenous antioxidant defense systems, potentially via activation of the NRF2 signaling pathway (25). However, the observed increase in DNA damage at 200 μg indicates that QA can exert pro-oxidant effects at higher concentrations. This observation is consistent with the biphasic, dose-dependent effects of natural antioxidants reported by Agarwal et al. (26).

MDA is a biomarker of lipid peroxidation, and its elevation reflects increased oxidative stress. GSH, a key intracellular antioxidant, remained largely unchanged across treatments, implying that GSH metabolism may be stable under cryopreservation conditions TAS reflects the cumulative activity of all plasma antioxidants. In contrast with our findings, several studies in the literature have reported that antioxidant supplementation reduces MDA levels in post-thaw ram (23) and goat (21) sperm. The addition of resveratrol has been associated with decreased MDA levels and enhanced antioxidant capacity in post-thaw ram semen (20). In the present study, TAS was increased and OSI remained statistically unchanged in the QA50–QA100, whereas the QA200 group exhibited elevated oxidant load and OSI levels. This pattern aligns with previous reports indicating that moderate antioxidant supplementation enhances TAS without inducing oxidative stress, while higher doses may increase the oxidant load, reflecting a dose-dependent biphasic effect (27, 28). In small ruminant studies, appropriately dosed plant-derived polyphenols have been shown to elevate TAS levels, which corresponds well with our QA100 findings (23). Our study did not observe any significant changes in GSH levels. Contradict to previous studies have reported that GSH supplementation can improve post-thaw sperm function, not all investigations have demonstrated a consistent increase in GSH; in some cases, GSH levels may vary in conjunction with local and enzymatic antioxidant parameters and are influenced by the measurement method or sampling time (29). The observed increases in MDA, TOS, and OSI in the QA200 group support the literature-reported phenomenon that some antioxidants can exhibit pro-oxidant behavior at high doses. This biphasic effect has been frequently documented in studies on phenolic antioxidants, indicating that a moderate dose of QA provides optimal efficacy while excessive doses should be avoided. The coexistence of increased motility-related or metabolic activity with elevated oxidative load in the QA200 group may reflect heightened mitochondrial activity leading to disproportionate ROS generation, which aligns with the biphasic response rather than a linear trend.

In light of the findings obtained that QA administration, particularly at QA100, significantly enhanced sperm viability, reduced lipid peroxidation, and increased HMMP which may be attributed to the stabilization of the plasma membrane or protection of membrane lipids against oxidative damage. These findings are generally consistent with previous reports on antioxidant supplementation in small ruminants; however, species-specific differences and variations in experimental protocols may influence the outcomes. Regarding sperm viability, numerous studies have reported that antioxidants such as phenolic compounds, quercetin and resveratrol added to semen extenders improve post-thaw sperm viability (10, 24). Conversely, QA200 resulted in decreased viability, likely reflecting dose-dependent effects associated with excessive oxidative stress (30). Regarding lipid peroxidation, previous studies have demonstrated that probes such as BODIPY-C11 can sensitively measure membrane lipid peroxidation following cryopreservation protocols, and antioxidant supplementation generally reduces lipid peroxidation levels. In the present study, QA at 50–100 μg/mL significantly decreased lipid peroxidation, indicating that QA can inhibit free radical chain reactions due to its antioxidant properties. Conversely, at the highest dose, lipid peroxidation increased, suggesting a potential pro-oxidant effect at elevated concentrations. The observed reduction in lipid peroxidation at QA100 is consistent with findings from small ruminant studies involving chlorogenic acid and other phenolic antioxidants, further supporting the antioxidant effect of QA (31). Regarding HMMP, previous studies have reported that antioxidant supplementation can enhance HMMP, with such increases generally correlating with ATP production and sperm motility. In the present study, QA100 elevated HMMP, suggesting enhanced energy production. Conversely, HMMP decreased at the QA200, which may indicate mitochondrial dysfunction associated with increased oxidative stress. The observed HMMP improvement at QA100 aligns with effects reported for Mito-TEMPO and similar agents in rams and other species, supporting a mitochondria-protective role of QA (32). Although the increase in HMMP in the QA100 group suggests improved mitochondrial function and ATP synthesis, an alternative explanation—mitochondrial hyperpolarization—should also be considered in spermatozoa (33). High HMMP is not always beneficial; it may indicate elevated metabolic activity without adequate ATP use (34) or increased ROS generation that leads to oxidative stress and reduced spermatozoa quality (35). However, a temporary rise in HMMP is essential for events such as fertilization (36, 37). Therefore, while the HMMP increase correlates with functional gains, future studies should include direct measurements of ROS production or ATP turnover rates to definitively confirm that this elevated mitochondrial potential reflects enhanced bioenergetic efficiency rather than detrimental hyperpolarization.

## 5 Conclusion

In conclusion, the findings of this study strongly demonstrate that QA possesses cellular protective potential during the cryopreservation of ram semen. The data indicate that particularly QA100 significantly improved sperm motility, certain kinematic parameters (VSL, VAP, and LIN), viability, and HMMP. This study demonstrates that QA, at appropriate doses, be able to preserve sperm DNA integrity after cryopreservation. Overall, QA at moderate doses represents a promising cryoprotective candidate, capable of preserving mitochondrial function, as well as plasma membrane integrity in spermatozoa. These findings are consistent with the biphasic redox behavior reported for phenolic compounds in the literature. QA provides encouraging evidence for the incorporation of natural phenolic compounds into semen cryopreservation protocols. Given that a statistical trend analysis was not conducted, the dose–response patterns identified in this study should be interpreted within the framework of a biphasic antioxidant–pro-oxidant effect rather than as a confirmed linear trend. Future studies should correlate QA’s effects with *in vivo* fertility outcomes and evaluate its performance under long-term storage conditions and across different small ruminant species to further clarify its practical application potential.

## Data Availability

The datasets presented in this study can be found in online repositories. The names of the repository/repositories and accession number(s) can be found in the article/supplementary material.
